# Echocardiographic predictors of futile recanalization in acute ischemic stroke

**DOI:** 10.1590/1806-9282.20250918

**Published:** 2025-12-15

**Authors:** Özgür Ertuğrul, Fırat Karaaslan, Reşit Yılmaz, Mehmet Cudi Tuncer

**Affiliations:** 1 University of Health Sciences, Gazi Yaşargil Training and Research Hospital, Department of Radiology – Diyarbakır, Turkey.; 2 University of Health Sciences, Gazi Yaşargil Training and Research Hospital, Department of Neurology – Diyarbakır, Turkey.; 3 Dicle University, Sur Campus, Faculty of Medicine, Department of Anatomy – Diyarbakır, Turkey.

**Keywords:** Acute ischemic stroke, Echocardiography, Thrombectomy, Endovascular procedures, Prognosis

## Abstract

**OBJECTIVE:**

The aim of the study was to investigate the relationship between echocardiographic parameters and clinical outcomes in acute ischemic stroke patients undergoing mechanical thrombectomy, aiming to identify predictors of poor prognosis despite successful recanalization.

**METHODS:**

This retrospective, single-center study included 320 acute ischemic stroke patients treated with mechanical thrombectomy for large vessel occlusion. Demographic, clinical, and echocardiographic data were collected. Univariable and multivariable logistic regression and receiver operating characteristic analyses were used. Futile recanalization was defined as achieving modified Thrombolysis in Cerebral Infarction 2b–3 with a poor functional outcome (mRS 3–6).

**RESULTS:**

Of the 320 patients, 176 (55%) were classified as favorable and 144 (45%) as futile recanalization. The futile group had a higher mean age (77.3±12.7 vs. 67.6±12.8; p<0.001), National Institutes of Health Stroke Scale score (median 17 vs. 14; p=0.007), and puncture-to-recanalization time (47.5 vs. 30 min; p=0.017), and lower Alberta Stroke Program Early Computed Tomography score (7.5 vs. 10; p<0.001). Echocardiographically, they had lower left ventricular ejection fraction (55 vs. 57.5%; p=0.036) and larger left ventricular end-diastolic diameter (4.8 vs. 4.5 cm; p=0.002). Multivariable analysis identified low Alberta Stroke Program Early Computed Tomography score (OR 0.192; p=0.002), high National Institutes of Health Stroke Scale (OR 1.212; p=0.029), and low left ventricular ejection fraction (OR 0.919; p=0.047) as independent predictors. Alberta Stroke Program Early Computed Tomography had the highest predictive value (area under the curve : 0.851), followed by left ventricular ejection fraction (area under the curve: 0.631), while National Institutes of Health Stroke Scale showed lower predictive power (area under the curve: 0.326).

**CONCLUSION:**

Poor outcomes after mechanical thrombectomy are associated with low Alberta Stroke Program Early Computed Tomography scores, high National Institutes of Health Stroke Scale, and reduced left ventricular ejection fraction. Echocardiographic evaluation, particularly of left ventricular ejection fraction, may aid in prognostication and treatment planning in acute ischemic stroke.

## INTRODUCTION

Acute ischemic stroke is one of the most devastating neurological disorders and a leading cause of disability and mortality worldwide^1^. Recent advances in acute ischemic stroke (AIS) management, particularly reperfusion therapies such as intravenous thrombolysis and mechanical thrombectomy (MT), have significantly reduced case fatality rates and improved post-stroke functional outcomes^2^. Despite achieving approximately 90% successful recanalization rates with MT, it is well recognized that clinical outcomes and prognosis do not always align with this technical success^3^. This discrepancy between high recanalization rates and suboptimal clinical outcomes points to a multifactorial recovery process that extends beyond vessel patency. Furthermore, the risk of complications associated with MT may adversely affect prognosis, rendering this life-saving intervention a double-edged sword^4^.

Cardiovascular events are common in stroke patients and represent a major cause of poor outcomes^5^. It has been suggested that the close interaction between the brain and heart, mediated by the autonomic nervous system and systemic inflammation, may contribute to a higher risk of cardiovascular complications in these patients^6^. Echocardiography, as a fundamental imaging modality for evaluating cardiac structure and function, provides valuable insights into cardiogenic factors that influence prognosis in AIS patients. Previous studies have demonstrated that cardiac measurements may be associated with stroke severity and can serve as prognostic indicators. Structural changes and functional impairments in the heart have emerged as independent risk factors for the development and progression of AIS^7^.

In one study, AIS patients receiving intravenous thrombolysis who had left ventricular systolic dysfunction exhibited significantly worse prognoses^8^. Obokata et al. further discovered that AIS patients with lower left atrial reservoir circumferential strain had higher mortality rates^9^. Additionally, another study reported that the presence of aortic valve sclerosis among various echocardiographic parameters was an independent predictor of poorer outcomes in AIS patients^10^.

In light of this evidence, a more comprehensive investigation into the prognostic value of echocardiographic parameters in AIS patients undergoing MT is crucial for optimizing treatment strategies and improving patient outcomes. This study aims to explore the impact of echocardiographic parameters on prognosis and to elucidate their potential associations with clinical outcomes in AIS patients treated with MT.

## METHODS

### Study design and ethical approval

This retrospective single-center study included adults (>18 years) with large vessel occlusion treated with MT at Diyarbakır Gazi Yaşargil Training and Research Hospital. Patients with missing data were excluded. The study was approved by the Clinical Research Ethics Committee (Approval No: 379) and conducted in accordance with the Declaration of Helsinki. Informed consent was not required due to the retrospective design.

### Patient selection and data collection

Demographic and clinical data, including age, sex, and medical history (e.g., hypertension, diabetes mellitus, atrial fibrillation, hyperlipidemia, coronary artery disease, peripheral artery disease, heart failure, previous stroke, malignancy, smoking, and alcohol use), were retrieved from electronic medical records. Patients who lacked complete transthoracic echocardiographic data within 72 h following MT were excluded from the final analysis.

### Neurological and radiological assessment

Neurological severity at admission was evaluated using National Institutes of Health Stroke Scale (NIHSS). Early ischemic changes were assessed via non-contrast cranial computed tomography (CT) using the Alberta Stroke Program Early CT Score (ASPECTS).

### Mechanical thrombectomy parameters

Data included occlusion site (e.g., MCA M1/M2, internal carotid artery [ICA] T/L, basilar artery, posterior cerebral artery [PCA], tandem lesions), MT technique (Solumbra, a direct aspiration first pass technique [ADAPT], or combined), number of device passes, final thrombolysis in cerebral infarction (TICI) score, and post-procedural intracranial hemorrhage. Time from symptom onset to femoral puncture and from puncture to recanalization was recorded. Intravenous thrombolysis and antiplatelet/anticoagulant use were also documented.

### Clinical outcome evaluation

Clinical outcomes were assessed at 3 months post-procedure using the modified Rankin Scale (mRS). Patients with mRS ≤2 were categorized as having favorable recanalization; those with mRS>2 were classified as having futile recanalization. Follow-up was conducted through outpatient visits or telephone interviews.

### Echocardiographic assessment

Transthoracic echocardiography (TTE) within 72 h post-MT was retrospectively analyzed. Parameters included interventricular septal and posterior wall thicknesses, left ventricular end-systolic diameter, left ventricular end-diastolic diameter (LVDd), and lower left ventricular ejection fraction (LVEF). Additional measurements covered the aortic root, ascending aorta, atrial and right ventricular diameters, and pulmonary artery diameter. Left ventricular hypertrophy was assessed based on wall thickness and clinical evaluation.

### Valvular assessment

Mitral and aortic valves were classified as normal, degenerative, calcific, or rheumatic based on TTE reports. Presence and semi-quantitative severity of mitral and aortic regurgitation were also recorded.

### Statistical analysis

Data were analyzed using IBM SPSS Statistics v25.0. Continuous variables were expressed as mean±SD (standard deviation) or median (IQR [interquartile range]), and categorical variables as counts and percentages. Group comparisons used chi-square, independent t-test, or Mann-Whitney U test, as appropriate. Univariable logistic regression identified predictors of futile recanalization; variables with p<0.05 were entered into the multivariable model. Receiver operating characteristic (ROC) analysis assessed predictive performance, with area under the curve (AUC) calculated for ASPECT score, LVEF, and NIHSS. The Hosmer-Lemeshow goodness-of-fit test was used to assess model calibration and yielded a non-significant result (p=0.412), indicating good model fit.

## RESULTS

### Patient distribution and baseline clinical differences

Baseline characteristics are shown in Table 1. Among 320 patients, 176 (55%) had favorable and 144 (45%) had futile recanalization. The futile group was older (77.3±12.7 vs. 67.6±12.8 years; p<0.001), had higher NIHSS scores (median 17, IQR: 14–19.75 vs. 14, IQR: 10–17.5; p=0.007), and lower ASPECT scores (median 7.5, IQR: 7–8 vs. 10, IQR: 9–10; p<0.001).

**Table 1:** Comparison of key demographic, clinical, radiological, and procedural characteristics between patients with favorable and futile recanalization following mechanical thrombectomy.

Variable		Favorable recanalization (n=176)	Futile recanalization (n=144)	p-value
Age, mean±SD		67.61±12.83	77.31±12.27	0.001*
Age ≥80 years, n (%)		56 (31.8%)	84 (58.3%)	<0.001*
Female, n (%)		100 (56.8%)	88 (61.1%)	0.820
Male, n (%)		76 (43.2%)	56 (38.9%)	
NIHSS, median (IQR)		14 (10–17.5)	17 (14–19.75)	0.007*
NIHSS ≥15, n (%)		67 (38.1%)	104 (72.2%)	0.002*
ASPECT, median (IQR)		10 (9–10)	7.5 (7–8)	0.001*
Dense MCA sign		88 (50.0%)	80 (55.6%)	0.658
Symptom-to-puncture time (min), mean±SD		241.81±116.39	272.63±109.44	0.210
Puncture-to-recanalization Time (min), median (IQR)		30 (21.25–48.75)	47.5 (30–70)	0.017*
Antiplatelet use		32 (18.2%)	44 (30.6%)	0.291
Anticoagulant use		36 (20.5%)	12 (8.3%)	0.208
Stroke type				
	Cardioembolism	100 (56.8%)	88 (61.1%)	0.751
	Large artery atherosclerosis	36 (20.5%)	28 (19.4%)	
	Other	40 (22.7%)	28 (19.4%)	
Occlusion site				
	MCA M1	84 (47.7%)	80 (55.6%)	0.304
	MCA M2	24 (13.6%)	20 (13.9%)	
	ICA T/L	20 (11.4%)	24 (16.7%)	
	Basilar	24 (13.6%)	4 (2.8%)	
	PCA	4 (2.3%)	4 (2.8%)	
	Tandem	20 (11.4%)	12 (8.3%)	
TICI score				
2b; 2c; 3		20 (11.4%); 48 (27.3%); 108 (61.4%)	40 (27.8%); 48 (33.3%); 56 (38.9%)	0.026*
Thrombectomy technique				
	Solumbra	144 (81.8%)	124 (86.1%)	0.680
	ADAPT	12 (6.8%)	8 (5.6%)	
	Solumbra+ADAPT	20 (11.4%)	12 (8.3%)	
	Number of passes, median (IQR)	1 (1–2)	2 (1–3)	0.013*
	Post-MT intracranial hemorrhage	28 (15.9%)	80 (55.6%)	0.001*

* Statistically significant differences (p<0.05) are marked with an asterisk.

TICI: thrombolysis in cerebral infarction. NIHSS: National Institutes of Health Stroke Scale; IQR: interquartile range; ASPECT: Alberta Stroke Program Early Computed Tomography; SD: standard deviation; MCA: middle cerebral artery.

Malignancy history was more common in the futile group (11.1 vs. 0%; p=0.037), and puncture-to-recanalization time was longer (47.5 min, IQR: 30–70 vs. 30 min, IQR: 21.25–48.75; p=0.017). No significant differences were found in sex, hypertension, or diabetes. Favorable outcomes were associated with higher TICI 2b/3 scores (p=0.026), whereas postprocedural intracranial hemorrhage was more frequent in the futile group (55.6 vs. 15.9%; p<0.001).

### Echocardiographic findings

The favorable group had higher LVEF (median 57.5%, IQR: 55–60 vs. 55%, IQR: 40–60; p=0.036) and smaller LVDd (4.5 cm, IQR: 4.4–4.875 vs. 4.8 cm, IQR: 4.6–5.15; p=0.002). Other echocardiographic parameters showed no significant differences (p>0.05).

### Univariable logistic regression analysis

Significant predictors of futile recanalization included:

- Older age (OR 1.067, 95%CI 1.023–1.113, p=0.003)
- Higher NIHSS (OR 1.13, 95%CI 1.03–1.25, p=0.013)
- Lower ASPECT score (OR 0.28, 95%CI 0.16–0.47, p=0.001)
- Longer puncture-to-recanalization time (OR 1.02, 95%CI 1.00–1.04, p=0.030)
- More device passes (OR 1.941, 95%CI 1.169–3.223, p=0.010)
- Postprocedural hemorrhage (OR 0.151, 95%CI 0.053–0.429, p=0.001)
- Larger LVDd (OR 3.108, 95%CI 1.222–7.902, p=0.017)
- Lower LVEF (OR 0.936, 95%CI 0.889–0.986, p=0.013)
- TICI score (OR 3.857, 95%CI 1.102–13.498, p=0.035)

### Multivariable logistic regression analysis

Independent predictors of futile recanalization were:

- Lower ASPECT score (OR 0.192, 95%CI 0.066–0.559, p=0.002)
- Higher NIHSS score (OR 1.212, 95%CI 0.985–1.492, p=0.029)
- Lower LVEF (OR 0.919, 95%CI 0.794–1.064, p=0.047)

These findings indicate that lower ASPECT scores, higher NIHSS, and reduced LVEF are independently associated with poor outcomes despite successful recanalization.

### Receiver operating characteristic analysis

**Figure 1:**
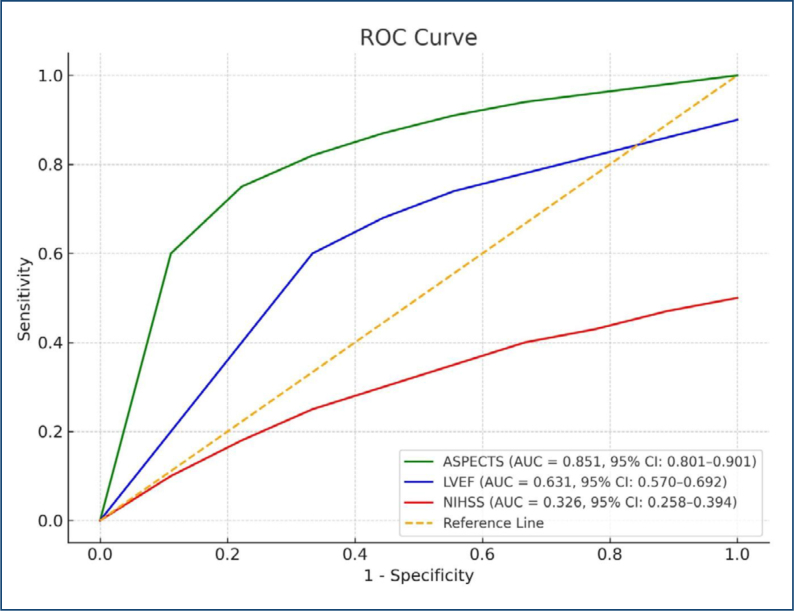
Receiver operating characteristic curve analysis for predicting clinical outcome. This figure presents the receiver operating characteristic curves for three variables: Alberta Stroke Program Early Computed Tomography score (green), left ventricular ejection fraction (blue), and National Institutes of Health Stroke Scale (red), in predicting clinical outcomes. Alberta Stroke Program Early Computed Tomography score shows the highest predictive value with an area under the curve of 0.851 (95%CI 0.801–0.901), indicating excellent discrimination. Left ventricular ejection fraction demonstrates moderate predictive ability with an area under the curve of 0.631 (95%CI 0.570–0.692). National Institutes of Health Stroke Scale shows poor performance with an area under the curve of 0.326 (95%CI 0.258–0.394), suggesting inverse or non-discriminative power. The orange dashed line indicates the reference line (area under the curve=0.5), representing no predictive capability. Among the tested parameters, Alberta Stroke Program Early Computed Tomography score was the most effective.

Predictive performance is shown in Table 2. The ASPECT score had the highest AUC (0.851, 95%CI 0.762–0.940, p=0.001), followed by LVEF (AUC: 0.631, 95%CI 0.506–0.755, p=0.045). NIHSS had lower predictive ability (AUC: 0.326, 95%CI 0.209–0.443, p=0.008). As illustrated in Figure 1, ASPECT had the strongest discriminative value.

**Table 2:** Univariate and multivariate logistic regression analysis of factors associated with futile recanalization after mechanical thrombectomy.

Variable	Univariate analysis		Multivariate analysis	
	OR (95%CI)	p-value	OR (95%CI)	p-value
Age	1.067 (1.023–1.113)	0.003		
NIHSS	1.13 (1.03–1.25)	0.013	1.212 (0.985–1.492)	0.029*
ASPECT	0.28 (0.16–0.47)	0.001	0.192 (0.066–0.559)	0.002*
Puncture-to-recanalization time (min)	1.02 (1.00–1.04)	0.030		
TICI score	3.857 (1.102–13.498)	0.035		
Number of passes	1.941 (1.169–3.223)	0.010		
Post-MT intracranial hemorrhages	0.151 (0.053–0.429)	0.001		
LVDd	3.108 (1.222–7.902)	0.017		
LVEF	0.936 (0.889–0.986)	0.013	919 (0.794–1.064)	0.047*

(*) Statistically significant associations (p<0.05) are marked with an asterisk.

OR: odds ratio; CI: confidence interval. NIHSS: National Institutes of Health Stroke Scale; ASPECT: Alberta Stroke Program Early Computed Tomography; LVDd: left ventricular end-diastolic diameter; LVEF: left ventricular ejection fraction.

## DISCUSSION

This study evaluated the prognostic value of echocardiographic parameters in acute ischemic stroke patients who underwent successful MT but had poor clinical outcomes. The findings emphasize that vessel patency alone does not guarantee neurological recovery, and cardiac function is a key determinant of post-stroke prognosis. According to the latest 2024 American Heart Association/American Stroke Association guidelines, early risk stratification after MT remains a critical challenge in AIS care^11^. While mRS >2 was used to define futile recanalization, further stratification into mRS 3–6 subgroups (e.g., moderate disability versus death) may have provided additional granularity in outcome assessment but was limited by statistical power considerations.

Multiple clinical and procedural variables were associated with futile recanalization. These include older age, lower ASPECT scores, higher NIHSS scores at admission, longer puncture-to-recanalization intervals, lower TICI scores, more device passes, and the occurrence of post-procedural intracranial hemorrhage. These factors have also been reported in previous studies as predictors of poor outcomes^12–14^. Delayed reperfusion may lead to irreversible ischemic damage, while repeated thrombectomy attempts can increase the risk of endothelial injury and distal embolization^15–17^.

Reduced LVEF was independently associated with worse clinical outcomes despite successful recanalization. Although TTE was performed within 72 h after MT as part of routine care, the exact timing varied slightly among patients. Transient cardiac dysfunction, such as neurogenic myocardial stunning, may have influenced echocardiographic measurements, and timing consistency between groups could not be fully verified retrospectively. Lower LVEF reflects impaired cardiac output, which may compromise cerebral perfusion during the critical recovery phase and impair neuronal survival and repair. Previousstudies have similarly reported that reduced ejection fraction is associated with unfavorable outcomes in acute ischemic stroke, including in patients undergoing thrombolysis or endovascular treatment^18,19^. In our study, although LVEF emerged as an independent predictor in multivariable analysis, its standalone discriminative power was modest (AUC: 0.631). Therefore, its prognostic value may be enhanced when integrated into a composite risk model alongside clinical and radiological parameters such as NIHSS and ASPECTS scores. Although our study did not specifically analyze outcome prediction based on categorical cut-offs, previous literature and clinical practice often consider LVEF values below 55% as indicative of systolic dysfunction and ASPECTS scores of 8 or less as markers of increased ischemic burden and poorer prognosis. In this context, patients presenting with both LVEF <55% and ASPECTS ≤8 may represent a particularly high-risk subgroup for unfavorable outcomes despite successful recanalization. Such patients could benefit from intensified post-thrombectomy cardiac monitoring, early cardiology consultation, and individualized hemodynamic optimization. Future multicenter studies are warranted to validate these thresholds within integrated risk prediction models. We focused on core systolic function metrics such as LVEF and LVDd, which were consistently available across all patients. However, other potentially informative parameters, including diastolic function indices, global longitudinal strain, and standardized assessments of valvular pathology were not routinely documented in TTE reports and thus could not be analyzed. Nonetheless, our findings suggest that systolic function assessment may contribute to early risk stratification and guide post-stroke care planning. Recent literature has also highlighted the prognostic significance of left ventricular dysfunction in acute ischemic stroke. In particular, Zhang et al. demonstrated that reduced LVEF measured within 24 h after intravenous thrombolysis was independently associated with poor 90-day outcomes in AIS patients, with a clear linear dose-response relationship^8^. Their findings corroborate our observations that impaired systolic function, even in the setting of successful recanalization, may compromise cerebral perfusion and hinder recovery. While their cohort focused on thrombolysis, our study extends this concept to patients undergoing MT, further emphasizing the role of cardiac output as a key determinant of neurological outcomes. These parallels reinforce the growing consensus that cardiac parameters, especially LVEF, should be considered in post-stroke risk stratification. Given that no single variable demonstrated sufficient predictive power in our cohort, we propose that future research should aim to develop a comprehensive risk stratification tool that incorporates echocardiographic markers such as LVEF, neurological assessments such as NIHSS, and imaging scores such as ASPECTS. Such a multiparametric model may improve individualized prognostication and inform targeted post-revascularization care strategies.

In the ROC analysis, ASPECTS showed the highest predictive value (AUC: 0.851), whereas LVEF had a moderate discriminative ability (AUC: 0.631). NIHSS demonstrated limited predictive power. These findings suggest that no single parameter may be sufficient for prognosis, and a composite approach integrating clinical, radiological, and cardiac variables may offer enhanced predictive accuracy.

Diastolic dysfunction, which reduces compliance and impairs filling, leading to reduced cardiac output. It is often observed in elderly patients and those with hypertension or atrial fibrillation, which are common comorbidities in stroke populations^20–22^. Reduced diastolic function may lead to insufficient cerebral perfusion, particularly when autoregulation is impaired.

Additional mechanisms may further explain the impact of cardiac dysfunction on stroke recovery. These include myocardial inflammation, impaired endothelial function, and reduced nitric oxide availability, all of which have been implicated in the pathophysiology of heart failure with preserved ejection fraction^23–25^. These systemic disturbances may impair cerebrovascular regulation and contribute to poor neurological outcomes.

This study has several limitations. Its retrospective design limits the ability to establish causal relationships. Variability may have arisen due to the non-standardized timing of echocardiographic assessments. As a single-center study, its generalizability to broader populations is restricted. In particular, regional differences in patient selection protocols, thrombectomy techniques, and peri-procedural care may influence outcomes and limit external applicability. Multicenter validation is needed to confirm our findings across diverse populations. Furthermore, cerebral perfusion was not directly evaluated with advanced imaging techniques, requiring indirect interpretation of the link between cardiac output and brain tissue perfusion. Infarct volume was also not routinely quantified, which may represent an important unmeasured confounder influencing clinical outcomes. Although the overall cohort size was adequate for primary analyses, subgroup analyses such as those stratified by occlusion site or thrombectomy technique may have been underpowered to detect statistically significant associations. Moreover, data regarding pre-stroke disability (pre-morbid mRS) and frailty status were not systematically available in the medical records, which may have introduced unmeasured confounding, as pre-existing disability could independently influence post-stroke functional outcomes. Additionally, the exclusion of patients with incomplete echocardiographic data may have introduced selection bias.

## CONCLUSION

This study shows that reduced LVEF is an independent predictor of poor outcomes in acute ischemic stroke patients undergoing MT. Although LVEF demonstrated statistical significance in multivariable analysis, its discriminative performance was modest (AUC: 0.631), indicating limited standalone utility. Therefore, LVEF should not be interpreted in isolation but rather considered as part of a composite risk assessment model that integrates clinical and radiological parameters such as NIHSS and ASPECTS. Incorporating cardiac function into early post-stroke risk stratification may help guide prognosis and clinical management following successful recanalization.

## Data Availability

The datasets generated and/or analyzed during the current study are available from the corresponding author upon reasonable request.
